# The effectiveness of visual-based interventions on health literacy in health care: a systematic review and meta-analysis

**DOI:** 10.1186/s12913-024-11138-1

**Published:** 2024-06-11

**Authors:** Elisa Galmarini, Laura Marciano, Peter Johannes Schulz

**Affiliations:** 1 Faculty of Communication, Culture & Society, Università Della Svizzera Italiana (University of Lugano), Lab, Office 201 (Level 2), Via Buffi 13, 6900 Lugano, Switzerland; 2 Harvard T.H. Chan School of Public Health and Dana Farber Cancer Institute, Boston, MA USA; 3 Department of Communication & Media, Ewha Womans University, Seoul, South Korea

**Keywords:** Health literacy, Visual-based intervention, Videos, Health knowledge, Review, Meta-analysis

## Abstract

**Background:**

Limited Health Literacy (HL) is an obstacle to accessing and receiving optimal health care and negatively impacts patients’ quality of life, thus making it an urgent issue in the health care system. Visual-based interventions are a promising strategy to improve HL through the use of visual aids and pictorial materials to explain health-related concepts. However, a comprehensive summary of the literature on the topic is still scarce.

**Methods:**

To fill this gap, we carried out a systematic review and meta-analysis with the aim to determine the effectiveness of visual-based interventions in improving comprehension of health related material in the clinical population. Independent studies evaluating the effectiveness of visual-based interventions on adults (> 18 years) and whose primary outcome was either health literacy (HL) or comprehension were eligible for the review. After a systematic literature search was carried out in five databases, 28 studies met the inclusion criteria and thus were included. Most of the studies were randomized controlled trials and they focused on HL and health knowledge as outcomes.

**Results:**

The review and meta-analysis showed that visual-based interventions were most effective in enhancing the comprehension of health-related material compared to traditional methods. According to meta-analytic results, videos are more effective than traditional methods (Z = 5.45, 95% CI [0.35, 0.75], *p* < 0.00001) and than the employment of written material (Z = 7.59, 95% CI [0.48, 0.82], *p* < 0.00001). Despite this, no significant difference was found between video and oral discussion (Z = 1.70, 95% CI [-0.46, 0.53], *p* = 0.09).

**Conclusions:**

We conclude that visual-based interventions, particularly the ones using videos, are effective for improving HL and the comprehension of health-related material.

**Supplementary Information:**

The online version contains supplementary material available at 10.1186/s12913-024-11138-1.

## Background

Health literacy (HL) is defined as “the degree to which individuals have the capacity to obtain, process, and understand basic health information and services needed to make appropriate health decisions” [1]. Limited HL has been described as an obstacle to accessing healthcare [2] and it negatively impacts the patient’s quality of life and health outcomes. Hence, it represents an urgent issue in the health care system. Prevalence of low HL levels are still high in Europe where 33% to 50% of people cannot comprehend basic health information [3]. This percentage increases in the United States, where only 12% of people shows a proficient level of HL [4, 5]. On the other hand, the prevalence of limited HL in Southeast Asian countries floats from 35.1% to 75.6%, with an average of 55.3% [6]. Limited HL negatively affects the healthcare system as a whole, including the use of resources and the economic burden. For example, limited HL is associated with non-adherence to pre-operative guidance and leads to detrimental health behaviors, including unhealthy lifestyles, drug abuse, and tobacco smoking [7, 8]. Berkman and colleagues [9] summarized the results of 96 studies and reported that limited HL is consistently associated with higher hospitalization rates, indiscriminate usage of the emergency department, low medication adherence, and failure to use preventive medicine services (i.e., cancer screening and vaccines). Limited HL often results in the misuse of resources and, consequently, wastes economic assets. For example, in the systematic review by Eichler and colleagues [10], summarizing ten studies, the costs associated with limited HL represented 3–5% of the overall expenditure covered by the healthcare systems in Switzerland and the United States. According to the authors, an individual with poor HL incurs an additional health expense ranging between $143 and $7.798 each year compared to a person with adequate HL. Hence, due to the high prevalence of limited HL in the population and its impact on societies and healthcare costs at large, there is an increasing need to address this gap by using more efficient methods to adapt medical materials to patients’ needs and abilities and, hence, to improve their knowledge.

In this regard, visual-based interventions are encouraging approaches to address limited HL due to their applicability and promising results [11]. The Centers for Disease Control and Prevention defines these interventions as “images, videos, and similar tools used to communicate information about a specific topic and to simplify the comprehension process” [12]. The advantages of using visual-based tools have already been documented in 1971, when Allan Paivio elaborated the Dual Coding Theory [13, 14] according to which the double encoding procedures reinforce the stimulus generated by visual cues. Images are encoded via multiple pathways, as they are simultaneously processed by the sensory and the verbal systems. In contrast, written words are only encoded by the verbal system. Since multiple systems are involved in elaborating visual materials, the “picture superiority effect” has been a key in facilitating comprehension and understanding [13, 14]. A qualitative synthesis performed in 2006 revealed that pictorial aids effectively enhanced patients’ comprehension of medications and treatments [15].

Furthermore, a systematic review of 52 studies reported significant improvements in understanding health-related materials among patients provided with audio-visual cues compared to standard methods like written information sheets or oral discussions with physicians [16]. However, this latter review focused on a broad range of interventions rather than visual-based ones. Furthermore, Lee and colleagues [17] reported that using icons, color codes, and larger font sizes improved health outcomes regarding hypertension, heart failure, and hypercholesterolemia among patients with limited HL. More recently, a scoping review was mapped the existing studies on digital video interventions for mental health literacy among young people, underlying the importance of studying these interventions and the initial stage in mapping the literature [18].

Although the literature provides a consistent number of independent studies on the effectiveness of visual-based intervention in improving the comprehension of health-related materials, systematic reviews on this topic are still scarce, while a meta-analysis is entirely lacking. Hence, the present study aims to systematically and meta-analytically summarize the scientific literature on the effectiveness of visual-based interventions in improving HL and the comprehension of health-related information in the adult population. Based on this contextual information, EG, LM and PS, formulated the research question in PICO format: In an adult population (P), composed of either healthy or not impaired individuals (P), what is the effect of the usage of visual-based intervention (I) in improving comprehension of health related materials (O) compared to standard methods of information delivery (C), through a systematic review and metanalysis investigating the effectiveness of these intervention (S). In doing so, we hypothesized that (i) visual-based interventions would effectively improve HL and the comprehension of health-related materials among adult patients. Also, we expect that (ii) patients who receive health information through more interactive material (e.g., videos) would report higher levels of comprehension than standard methods of information delivery (e.g., oral discussion or written information).

## Methods

The systematic review and meta-analysis was performed according to current PRISMA guidelines [19]. EG, LM and PS determined the most appropriate databases to conduct the literature search. Various databases were evaluated in order to ensure comprehensive coverage of relevant literature. As a result, five databases were used to perform a literature search on March 22nd: Behavioral Science Collection, CINHAL, Communication and Mass Media Complete, MEDLINE, and PsychInfo. EG and LM developed the list of keywords tailored to the research objectives. PS revised and approved them ensuring alignment with the study’s aim. The search terms covered two key topics: “visual intervention” and “health literacy”. The Boolean Operators “OR” and “AND” were used to divide keyterms within and between the two categories, respectively (see Table S1 in the Appendix for the complete list of keywords). Additionally, EG carried out a handsearch in Google Scholar on April 14th by entering selected keywords and reviewing the first 100 entries of each combination. EG performed the selection procedure between March 23rd and April 20th.

### Eligibility criteria

Studies were included in the systematic review and meta-analysis according to inclusion criteria previously defined by the authors. In particular, a study has to (1) be written in English and published in a peer-reviewed journal, (2) focus on visual-based intervention within the health-care setting, (3) include an adult population (≥ 18 years), (4) include either healthy or individuals from a clinical setting, and (5) use a measure of HL or comprehension of health related material as the outcome. No restriction was applied to the visual type or to the disease or healthcare area under investigation. Additionally, studies reporting a result convertible into an effect size were further included in the meta-analysis. A study was excluded if: (1) it investigated other interventions than visual aids, (2) the study population was composed of individuals under 18 years, (3) the study population consisted of adults on behalf of minors or impaired individuals, (4) HL or comprehension was not explicitly stated as outcomes, and (5) if the language differed from English. Duplicates, dissertations, books, magazines, reviews, editorial material, letters, and retracted publications were also excluded.

### Data collection

For each included publication, EG extrapolated the following information: title, author(s)’s name, year of publication, health-care area (that was further divided into health promotion, health prevention, disease management, and consent or risk management), disease under investigation, study design, geographical location, description of the setting, the final number of participants, sample characteristics (i.e., age, gender, ethnic group), type of visual-based intervention, comparators such as standard methods of information delivery (written information sheet, oral discussion with doctor), outcome measure, the definition of HL (if present), and a brief description of the results.

### Risk of bias assessment

The quality of the included studies was evaluated according to a selection of items from the Cochrane Risk of Bias assessment (RoB2) [20]. In particular, each article was evaluated for the procedure under which subjects were assigned to study arms (selection bias), the extent to which participants and researchers were aware of the allocation process (performance bias), the management of missing data (attrition bias) and the methods employed for measuring the outcome (detection bias) [20]. A single coder (EG) performed this assessment on Review Manager 5 (RevMan 5.4.1).

### Data analysis

The meta-analysis was performed on RevMan 5.4.1. EG, LM, and PS contributed to the analysis and interpretation of data. The team used a standardized mean difference to calculate the effect size. Cut-off levels of Hedge’s *g* were set at 0.2, 0.5, and 0.8 for small, medium, and large effect sizes, respectively [21]. The inconsistency index (I^2^) was used to measure heterogeneity across studies [21]. In order to interpret the results of the I^2^, the following standard cut-off values were set: an I^2^ comprised between 0 and 40% (low) represented no significant difference across studies, whereas an I^2^ greater than 75% indicated considerable heterogeneity; the interval in between was interpreted as either moderate or substantial differences between studies [21].

## Results

### Characteristics of included studies

**Fig. 1:**
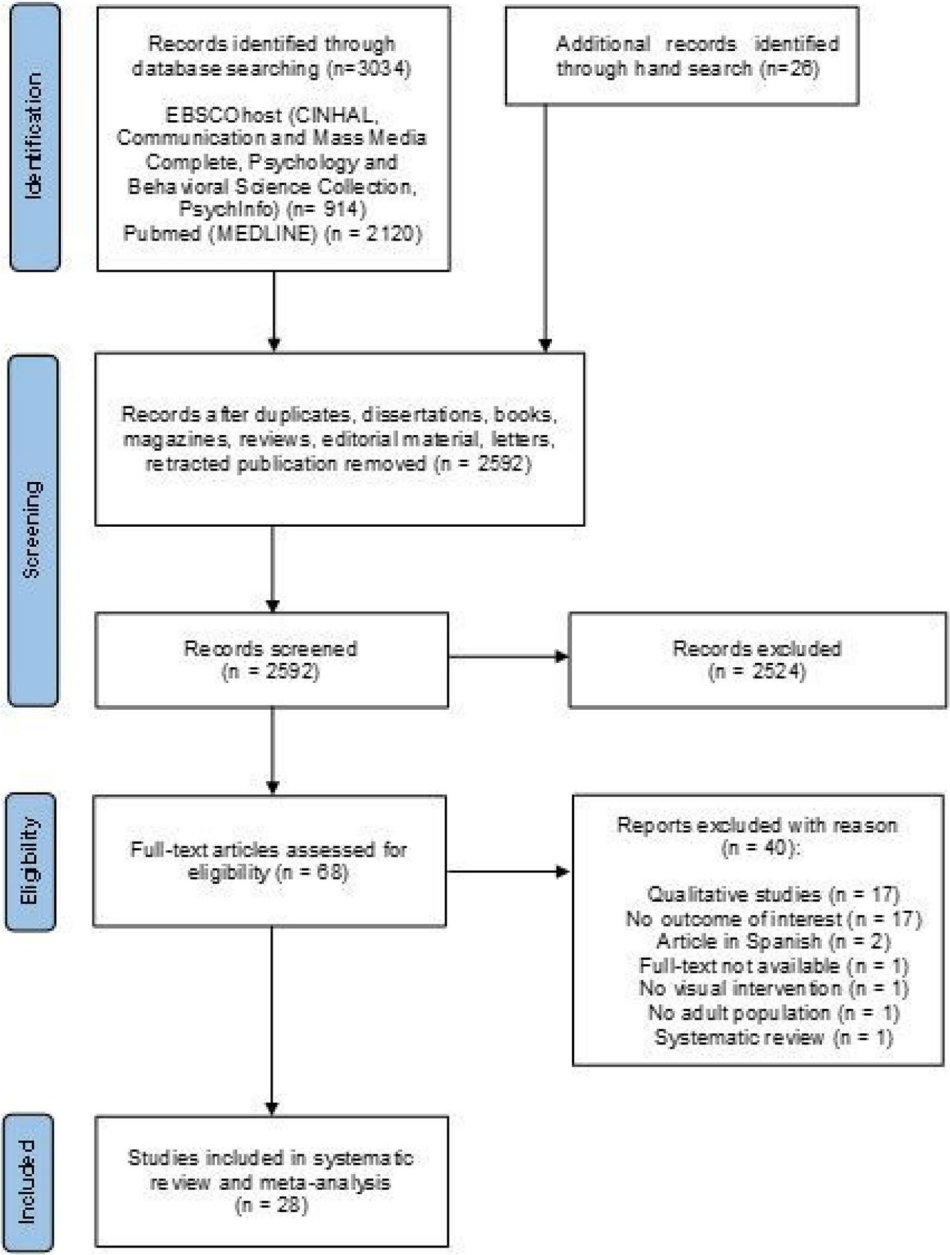
PRISMA flowchart

The literature search in the databases and hand search returned 3060 results. Out of these, 442 were duplicates, dissertations, books, and reviews and hence were excluded. Then, titles and abstracts of 2592 entries were screened, ending in 68 records eligible for full-text screening. Throughout the screening of titles and abstracts, a significant number of studies were deemed ineligible for inclusion. The predominant reasons for exclusion were the lack of measurement of the outcome of our interest, namely the comprehension of health related material and, more frequently, a predominant focus on ophthalmologic conditions or pathologies related to the visual system, a bias likely introduced by specific keywords within the search string pertaining to the domain of visual interventions. Among these, 28 studies met the inclusion criteria and were then included in the systematic review. Out of these, ten studies reported data convertible into effect sizes and were then included in the meta-analysis. The selection process is represented in the PRISMA flowchart (see Fig. 1).

### Description and design of included studies

The current systematic review is based on 28 studies [11, 22–48]. The characteristics of the included studies are reported in the Appendix (Table S2).

The total number of participants included in the studies was 5347, with sample sizes of individual studies ranging from 31 to 821 participants (Fig. S1). Included studies reported an equal distribution in terms of males (53.36%) and females (45.46%), whereas one study did not report gender information [45] (Fig. S2). The mean age of participants in the included studies was 53.8 ± 12.1 years, ranging from a minimum of 33.7 to a maximum of 74 years (Figs. S3 and S4). Only few studies provided information regarding participants’ educational level (*n* = 19) or ethnic group (*n* = 9). Among these, 50.7% had less than a high school degree, whereas 22.25% and 27.04% had a high school diploma or more, respectively (Fig. S5). Information regarding the ethnic group was available for 1451 participants, among which 50.52% were Caucasian, 17.44% were Hispanic, 25.78% were African-American, and 0.83% were Asian. The remaining 5.44% represented study participants for whom the ethnic group differed from those mentioned above (Fig. S6).

All the studies were randomized clinical trials (RCTs) except for three. Among the RCTs, two adopted a randomized cross-over study design [32, 48]. Whereas, within non-RCTs, two adopted a quasi-experimental design [29, 40] and one a pre-post study design [25] (Fig. S7).

The majority of the studies (*n* = 14) were conducted in North America (U.S. and Canada) and Oceania (*n* = 6). In contrast, fewer studies were conducted in Europe (*n* = 4), Asia (*n* = 3), and Africa (*n* = 1) (Figs. S8 and S9). The effectiveness of the visual-based intervention was evaluated on context-specific material; evaluated information covered topics such as informed consent (*n* = 18), health prevention (*n* = 8), and disease management (*n* = 2). Medical areas of interest were equally distributed among the following categories: “surgery” (*n* = 8) [28, 32, 36, 38, 41, 42, 44, 45], “cancer” (*n* = 8) [11, 25, 27, 29, 30, 34, 46] and “procedure” (*n* = 8) [22, 23, 26, 33, 37, 43, 47, 48]. The latter included colonoscopy, contrast administration, prostate vaporization, and urological and laser treatment, whereas cardiovascular diseases were investigated in four studies [24, 31, 35, 39].

### Description of visual-based interventions

The preferred format for visual-based intervention was the video (*n* = 20), followed by multimedia-based presentations (*n* = 3), graphs (*n* = 1), and a booklet combined with either a video (*n* = 2), a multimedia-based presentation (*n* = 1), or a mind map (*n* = 1). The types of visual cues and their frequencies are summarized in the Appendix (Table S3). The average length of the video, calculated on 17 studies, was 10.88 ± 7.347 min (ranging from 2 to 30 min). Information regarding the language used in the videos was provided only in two studies, including English, Spanish, and Kiswahili [25]. If not reported, a local or national language was likely used.

The reading level of the video material was provided in three studies, and it was equal to 8th [26, 43] or 7th grade [41]. Concerning the multimedia-based presentation of material, it was impossible to determine the material’s average length: two studies reported data with different units of measurement, minutes [28] or just the number of slides [32] whereas the others did not disclose any information regarding the length of the material. Similarly, the reading level was provided only in one study, which was 7th grade [32].

Likewise, it was not possible to provide aggregated data on the length and reading level of the booklet format. The units of measurement, reported only in two studies, were not homogeneous: 18 min [46], 12 pages [11], and 2407 words [30]. Eventually, a mind map (*n* = 1) and a graph (*n* = 1) were the least represented formats. However, there was a lack of informative data concerning these strategies.

### Description of outcome measures

Comprehension of health related material was the primary investigated outcome. However, this variable was measured differently across studies and included studies usually assessed patients’ level of comprehension through questionnaires explicitly developed for the study. Questionnaires were in the format of multiple choice (*n* = 10), true/false questions (*n* = 9), combined approach (multiple choice + true/false) (*n* = 2), and in some cases, computational questions, which required individuals to perform basic mathematical tasks regarding the health-related topic. However, it is worth noting that some studies (*n* = 6) did not disclose any information concerning the type of assessment. The individual’s level of comprehension was generally obtained by summing up the scores attributed to each correct answer. Occasionally, the final score was converted into a percentage. Furthermore, besides comprehension, three studies [11, 27, 47] measured participants’ level of HL with validated instruments: Test of Functional Health Literacy Ability – shortened (S-TOFHLA), Brief Health Literacy Scale (BHLS), Newest Vital Sign (NVS) and Rapid Estimate of Adult Literacy in Medicine (REALM) (Table S4).

### Results of the systematic review

Overall, visual-based interventions were generally described as an effective instrument for enhancing the comprehension of health-related material compared to traditional methods (e.g., written material). In particular, in fourteen studies out of 22 (20 video-only + 2 videos combined with booklet) that adopted video material, participants in the intervention group (video format) had significantly higher comprehension levels than those in the control group (traditional method). Among the remaining six studies, two reported improvements in the level of comprehension after exposure to both video and standard material. However, the difference between the study arms was still not significant.

Among the studies that adopted multimedia-based presentation (*n* = 3), two registered a significant difference between study arms and showed a higher comprehension level than the control group. Instead, no significant difference was found in a study evaluating the effectiveness of charts (bar graph). Eventually, among the studies evaluating the effectiveness of booklets (*n* = 4), only one reported that participants provided with such format had higher comprehension levels than those provided with either leaflets or videos.

### Meta-analytic results

Different meta-analyses were performed to compare video effectiveness to traditional information delivery methods. Due to the paucity of available data, studies including formats like multimedia-based presentations, charts, booklets, and mind maps were excluded. Also, among the studies adopting videos as a visual intervention tool (*n* = 20), some were removed due to the lack of data regarding mean and standard deviation (*n* = 9) or the study design (*n* = 1). As a result, ten studies were included in the meta-analyses for a total of 1784 participants. Three meta-analyses were performed in order to compare videos with (1) traditional methods, (2) written communication, and (3) verbal discussion with the doctor.

**Fig. 2:**
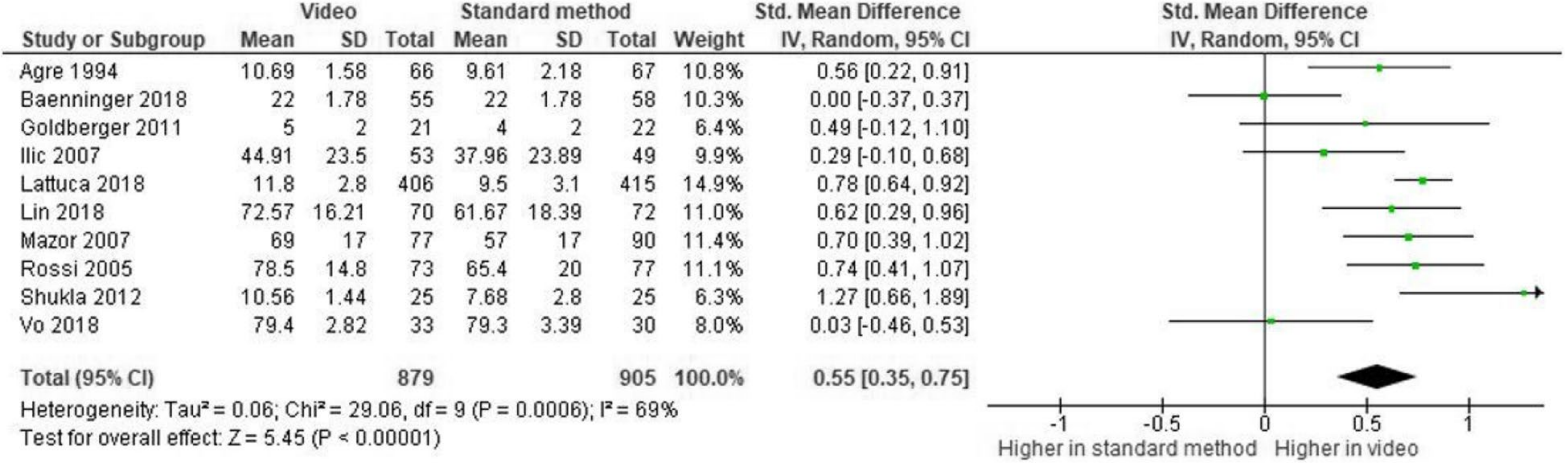
Meta-analysis: video vs traditional methods

The comprehension level was significantly higher among participants who received a video than those provided with traditional methods. In particular, a medium effect size was found when comparing video materials with the standard method of information delivery (without discrimination of the type) (k = 10, Hedge’s g = 0.55, 95% CI [0.35, 0.75], *p* < 0.001, see Fig. 2).

**Fig. 3:**
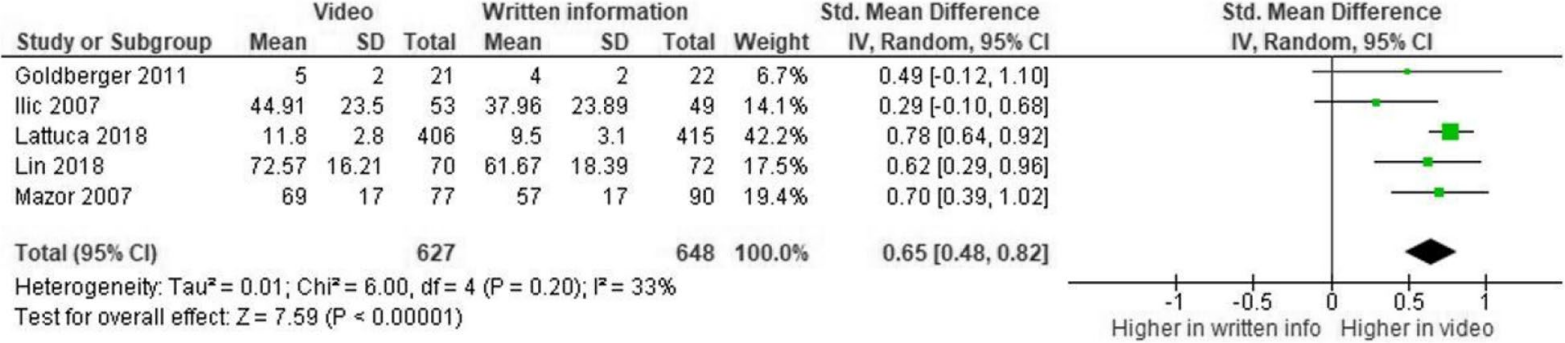
Meta-analysis: video vs. written information

Similarly, a medium effect size was also found in the case of videos compared to written information (k = 5, Hedge’s g = 0.65, 95% CI, [0.48, 0.82], *p* < 0.001, see Fig. 3).

**Fig. 4:**
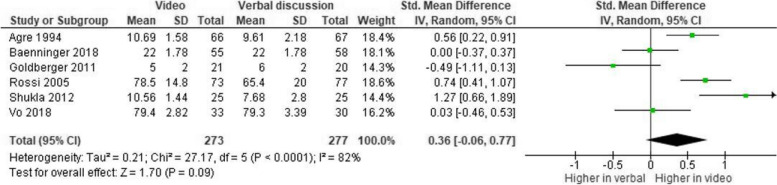
Meta-analysis: video vs. oral discussion

Conversely, no significant difference was found when videos were compared only to oral discussion with the doctor (k = 6, Hedge’s g = 0.36, 95% CI, [-0.06, 0.77], *p* = 0.09, see Fig. 4).

The level of heterogeneity was considerably high across the three meta-analyses. The former and the last meta-analysis reported high levels of inconsistency (*I*^2^ = 69%, *p* = 0.0006 and *I*^2^ = 82%, *p* < 0.0001). Instead, the inconsistency index was not significant in the second meta-analysis (*I*^2^ = 33%, *p* = 0.20). These results could be due to substantial differences in sample sizes, study design, the material used for the intervention, topic of interest, medical area investigated, and the use of different measures to assess patients’ levels of comprehension.

### Risk of bias

In general studies showed a low risk of bias, except for the selection bias, for which a small proportion of studies (*n* = 4) is stated to be unclear. This result is mainly due to the study design: two studies [29, 34] adopted a quasi-experimental approach and one a pre-post study design [25]. The remaining study was evaluated as medium risk of bias because of significant baseline differences between the study arms, which may indicate failure in the randomisation process. The good quality of the studies is mainly related to homogenous setting, the possibility to immediately assess the outcome soon after the intervention and the objectivity in the measurement, which avoided outcome assessors to provide a personal and subjective evaluation.

## Discussion

The current systematic review aims to evaluate the effectiveness of visual-based interventions to improve patients’ HL and levels of comprehension of health related material. To our knowledge, this is the first attempt to systematically and meta-analytically summarize evidence on the performance of these tools in the context of HL and knowledge of health information. According to qualitative results, visual-based interventions generally represented an effective way to improve comprehension of health-related information. However, their effectiveness mainly depends on the format employed. In particular, out of the 28 included studies, the most favorable evidence is provided by the studies that used videos as intervention tools (*n* = 20). More than half reported a significant increase in participants’ levels of comprehension.

Compared with traditional methods, participants provided with video material showed higher comprehension levels, with medium effect size, than those with standard oral or written material. In line with the Dual Coding Theory [14], the effectiveness of visual-based interventions is mainly due to their increased interactivity [49] and reduced reading efforts [50]. Indeed, conversely to traditional communication strategies, visual tools involve greater patient participation [51]. For instance, more interactivity is conveyed by quizzes and short games embedded in some visual tools, thus augmenting comprehension [52]. This is in line with previous research on the effect of interactivity on comprehension and attitude in the healthcare context [53], according to which the level of interactivity predicted patient comprehension. Even the absence of words, either partial or total, may have contributed to significant improvements in the outcome. Functional HL, a basic component of HL which includes basic skills of reading and numeracy [54], dramatically impacts an individual’s ability to understand and appraise health-related material. Indeed, visual cues require less effort to be processed and recalled than written information; this may make information more accessible and comprehensible to those with limited HL [50]. Eventually, the effectiveness of these instruments could be explained by referring to the Dual Coding Theory [14]. Our results further show that the “picture superiority effect” owned by visual cues allows distinguishing these tools from more traditional methods (i.e., written communication) since the multimedia content embedded into these tools (i.e., video, images, audio) was subjected to a double encoding process [14], which in turn allowed the stimulus to be consolidated, thus improving the comprehension and recall of the information.

However, the difference was no longer significant when the video format was confronted with an oral discussion with a physician. The lack of a significant difference between video and verbal discussion could be attributed to the benefits of face-to-face consultations, such as the possibility to interpret non-verbal messages [55] and directly assess patient’s comprehension level [56]. Non-verbal communication is essential in the doctor-patient interaction [57]. Thanks to the possibility of perceiving patients’ emotional states through the analysis of gestures, postures, and mimics, the physician can adjust the conversation to make the patient feel more confident with the information received in terms of content and form. In practice, the doctor may avoid complex medical terminology to reduce cognitive efforts on behalf of the patient and simplify the comprehension process [56, 58]. Similar strategies, like the “teach-back method” [56], involve the immediate assessment of the patient’s level of comprehension. Adopting such solutions might have noticeably reduced the gap in comprehension between the intervention (video) and control group (oral discussion), which eventually resulted in a non-significant difference. Hence, oral discussion with a doctor may represent a valuable and complementary intervention to be implemented for patients with low health literacy.

Finally, some remaining studies reported increases between pre-and post-intervention, though not significant. In particular, concerning multimedia-based presentations, two studies out of three registered a significant difference between the intervention and control groups. However, due to the low number of studies included in this category, it is not accurate to conclude that multimedia-based presentations are valuable tools for improving comprehension of medical material. Conversely, charts were ineffective. However, their use was investigated by only one study. Hence, future studies are needed to better explore the effectiveness of multimedia material in addition to the video.

Based on these findings, we encourage the adoption of visual tools for medical information purposes to set up innovative, effective, and fair communication. Nevertheless, we call for more research reporting more details on these interventions' characteristics, graphical layouts, and designs. Also, more research is needed regarding different medical areas and contexts, chronic and acute illnesses, and mental health problems.

### Limitations

The present study has some limitations. First, the lack of validated scales for measuring comprehension of medical material might have introduced biases in the analyses and augmented the heterogeneity of the meta-analytic results. Indeed, each of the included studies measured the outcome through different scales. The heterogeneity found could also be attributed to variations in sample size and participants’ characteristics, including age, gender, ethnicity, and educational level. Second, included studies were not equally distributed across the different types of visual interventions. As a result, we could not draw robust conclusions on the effectiveness of some tools (i.e., charts, booklets, and mind maps). Third, included studies were mainly from limited geographical regions. Only a few were conducted in low- and middle-income countries, where adopting such instruments may represent an effective tool to improve people’s well-being and state of health [59]. Moreover, the absence of studies from South America may represent a methodological and research bias, that may have significantly limited the landscape of the included studies. Additionally, our study might be limited due to the lack of consideration of specific terms related to interactive visual resources (e.g., video). Hence, we suggest that future studies should include detailed descriptions of visual- and video- based interventions.

Finally, we call for future research to develop specific comprehension scales to increase and facilitate comparability across studies. Moreover, more data are needed to compare the mere use of videos with the combination of video and oral discussion, since adopting this mixed approach is a promising strategy for improving, even more, the comprehension of medical material. Finally, it would be useful to investigate the effectiveness of visual-based intervention in different clinical settings and populations, including young people. Antelo and colleagues [60] investigated the health literacy of women in Argentina regarding Human Papilloma Virus (HPV) after using a mobile counseling app and found that a mobile app is a good tool to help HPV-positive women by providing information and reducing fears [60]. Lee and colleagues [61] analyzed the effect of user-centered mHealth intervention apps (e.g., *Click to Connect*, *PLANET MassCONECT,* and *SmartPhone App for Public Health*) to inform future work regarding app designing in Massachusetts and found that one of the critical features that increase HL among underserved communities is to design apps with usability, readability, and navigability in mind. Indeed, the vast majority of the studies in the review regarded the informed consent process, whereas publications on disease management and health prevention are still scarce. Regarding the need to investigate the effectiveness of similar instrument in other domains and population, Ito-Jaeger and collegues, in their scoping review on 17 studies, illuminated the effectiveness of digital video interventions as powerful tool in enhancing mental health literacy among young individuals [18].

## Conclusion

Visual-based interventions are effective tools for improving patients’ levels of comprehension. In particular, the adoption of video formats significantly augments comprehension compared to more traditional methods. However, no additional benefits were found compared to the oral discussion with a physician. Future studies should investigate whether the combination of these methods (visual-based intervention + oral discussions with a physician) is more effective than the simple provision of the video format.

### Supplementary Information

Supplementary Material 1.

## Data Availability

No datasets were generated or analysed during the current study.

## Abbreviations

HL: Health Literacy
S-TOFHLA: Test of Functional Health Literacy Ability Shortened
BHLS: Brief Health Literacy Scale
NVS: Newest Vital Sign
REALM: Rapid Estimate of Adult Literacy in Medicine
