# Supplementary material for: The effectiveness of visual-based interventions on health literacy in health care: a systematic review and meta-analysis
Source: BMC Health Serv Res. 2024 Jun 11;24:718. doi: 10.1186/s12913-024-11138-1 (PMC11165863; doi:10.1186/s12913-024-11138-1)
Supplement: Supplementary file 1 — Supplementary Material 1. [file 12913_2024_11138_MOESM1_ESM.docx]

**APPENDIX**

**Figure S1.** Sample size frequency distribution

**Figure S2.** Gender distribution

**Figure S3.** Age distribution


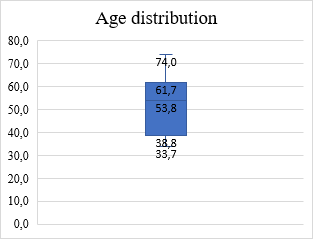


**Figure S4.** Age groups

**Figure S5.** The educational level of the participants

**Figure S6.** Ethnic group

**Figure S7.** Study design of the included publications

**Figure S8**. Geographical distribution of studies divided by continent or macro-area

**Figure S9.** Geographical distribution of studies divided by country

**Table S1.**  Example of search strategy on PUBMED

| 1. **Visual intervention** |
| --- |
| Visual based* OR visual communicate* OR visual prompt* OR visual cue* OR visual intervention* OR visual message* OR visual-based message* OR illustration* OR vignette* OR icon* |
| 1. **Health literacy** |
| Health literac* OR health numerac* OR REALM OR functional health* |
| **FINAL SEARCH STRATEGY: Visual intervention AND health literacy (n = 2120)** |
| **(**Visual based* OR visual communicate* OR visual prompt* OR visual cue* OR visual intervention* OR visual message* OR visual-based message* OR illustration* OR vignette* OR icon*) AND (Health literac* OR health numerac* OR REALM OR functional health*) |

**Table S2.** Study and sample characteristics of all studies retained in the systematic review (n=28)

| **Study (first author, publication year)** | **Study type** | **Country** | **Disease category** | **Sample size (n)** | **Gender (% female)** | **Age (M, SD)** | **Visual intervention** | **Comparator** |
| --- | --- | --- | --- | --- | --- | --- | --- | --- |
| Agre, 1994 | RCT | USA | Colonoscopy | 201 | 54.7% | 61 (range: 21-86) | Video | Oral discussion |
| Baenninger, 2018 | RCT | Switzerland | Oculist laser | 113 | 45.1% | 35.3 (9.6) | Video | Written information |
| Bowers, 2015 | RCT | Canada | Cardiovascular disease procedure | 93 | 37.6% | 60.9 (range: 19-89) | Video | Verbal information |
| Cooper, 2020 | Non-RCT | Tanzania | Cervical cancer | 764 | 100% | 36.4 (11.1) | Video | n.a. |
| Cowan, 2007 | RCT | USA | Contrast administration | 107 | 63.2% | 41.5 (16.3) | Video | Verbal information |
| Donelle, 2009 | Longitudinal | Canada | Cancer | 49 | 46.9% | 62.6 (n.a.) | Graph | Text |
| Ellet, 2014 | RCT | Australia | Gynecological surgery | 41 | 100% | 36.1 (n.a.) | Multimedia-based presentation | Verbal information |
| Frosch, 2008 | Quasi-experimental | USA | Prostate cancer  Colon cancer | 207 | 41.3% | 61.2 (n.a.) | Video | Brochure |
| Gattellari, 2015 | RCT | Australia | Prostate cancer | 409 | 0% | 57.7 (n.a.) | Video,  Booklet | Leaflet |
| Goldberger, 2011 | RCT | USA | Cardiovascular disease procedure | 62 | 28.6% | 61 (16) | Video, booklet | Verbal information |
| Gyomber, 2010 | Prospective randomized cross-over | Australia | Prostatectomy | 40 | 0% | 61 | Multimedia-based presentation | Written information |
| Ham, 2016 | RCT | Korea | Prostate vaporization | 40 | 0% | 66.9 (6.4) | Video | Written information |
| Housten, 2020 | RCT | USA | Colorectal cancer | 187 | 63.1% | 58.4 (n.a.) | Video,  Booklet | n.a. |
| Ilic, 2008 | RCT | Australia | Prostate cancer | 156 | 0% | 56.7 (n.a.) | Video | Pamphlet,  Internet |
| Lattuca, 2018 | Prospective trial | France | Angiography | 821 | 28% | 67.3 (11.6) | Video | Written information |
| Lin, 2018 | RCT | China | Trauma surgery | 142 | 44.4% | 37.1 (n.a.) | Video | Written information |
| Luck, 1999 | RCT | Australia | Colonoscopy | 150 | 48.6% | 53 (n.a.) | Video | Written information |
| Mason, 2003 | RCT | UK | Gynecological surgery | 31 | 100% | 33.7 (n.a.) | Video | Verbal information |
| Mazor, 2007 | RCT | USA | Cardiovascular disease medication | 326 | 36.5% | 66.1 (n.a.) | Video | Written information |
| Nagamma, 2020 | Quasi-experimental | India | Cervical cancer | 166 | 100% | 42.5 (2.5) | Video | Pamphlet |
| Rossi, 2004 | RCT | USA | Trauma surgery | 48 | 43.7% | 37.9 (n.a.) | Video | Verbal information |
| Rossi, 2005 | RCT | USA | Trauma surgery | 150 | 51.3% | 49 (n.a.) | Video | Verbal information |
| Shaw, 2001 | RCT | USA | Colonoscopy | 86 | 56% | 53.4 (n.a.) | Video | Standard |
| Shukla, 2012 | RCT | USA | Cataract surgery | 100 | 6% | 74 (n.a.) | Video | Verbal and written information |
| Vo, 2018 | RCT | USA | Cataract surgery | 63 | n.a. | n.a. | Video | Verbal information |
| Volk, 2008 | RCT | USA | Prostate cancer | 352 | 0% | 56.3 (n.a.) | Multimedia-based presentation, booklet | n.a. |
| Walker, 2007 | RCT | UK | Rheumatoid arthritis | 363 | 70.5% | 61.8 (n.a.) | Booklet,  mindmap | n.a. |
| Winter, 2017 | RCT cross-over | Australia | Urological procedure | 88 | 25% | 54 (n.a.) | Video | Verbal information |

**Table S3.** Frequency distribution of visual intervention strategies

| VISUAL INTERVENTION | FREQUENCY (n) |
| --- | --- |
| Video | 20 |
| Multimedia-based presentation | 3 |
| Graph | 1 |
| Booklet + video | 2 |
| Booklet + multimedia-based presentation | 1 |
| Booklet + mind map | 1 |
| TOTALE | N = 28 |

**Table S4.** Outcome measurement and characteristics

| **Study (first author, publication year)** | **Measure of health literacy** | **Type of assessment** | **N° of items** |
| --- | --- | --- | --- |
| Agre, 1994 | n.a. | n.a. | 13 |
| Baenninger, 2018 | n.a. | True/false | 25 |
| Bowers, 2015 | n.a. | True/false | 5 |
| Cooper, 2020 | n.a. | Multiple choice | 6 |
| Cowan, 2007 | n.a. | Multiple choice | 10 |
| Donelle, 2009 | NVS | Computation | 6 |
| Ellet, 2014 | n.a. | True/false | 14 |
| Frosch, 2008 | n.a. | True/False | n.a. |
| Gattellari, 2015 | n.a. | True/false + multiple choice | 14 |
| Goldberger, 2011 | n.a. | Multiple choice | 5 |
| Gyomber, 2010 | n.a. | Multiple choice | 26 |
| Ham, 2016 | n.a. | n.a. | 15 |
| Housten, 2020 | S-TOFHLA  BHLS | n.a. | 14 |
| Ilic, 2008 | n.a. | Multiple choice | 5 |
| Lattuca, 2018 | n.a. | n.a. | 16 |
| Lin, 2018 | n.a. | Multiple choice | 10 |
| Luck, 1999 | n.a. | n.a. | 12 |
| Mason, 2003 | n.a. | True/false | 20 |
| Mazor, 2007 | n.a. | True/false | 22 |
| Nagamma, 2020 | n.a. | n.a. | 9 |
| Rossi, 2004 | n.a. | Multiple choice | 12 |
| Rossi, 2005 | n.a. | Multiple choice | 15 |
| Shaw, 2001 | n.a. | True/false + multiple choice | 14 |
| Shukla, 2012 | n.a. | Multiple choice | 12 |
| Vo, 2018 | n.a. | True/false | 5 |
| Volk, 2008 | n.a. | True/false | n.a. |
| Walker, 2007 | REALM | True/false | 40 |
| Winter, 2017 | n.a. | Multiple choice | 28 |

Legend: S-TOFHLA= Short-Test of Functional Health Literacy in Adult; BHLS= Brief Health Literacy Scale; NVS= Newest Vital Sign; REALM=Rapid Estimate of Adult Literacy in Medicine
